# Novel Isoforms of Adhesion G Protein-Coupled Receptor B1 (ADGRB1/BAI1) Generated from an Alternative Promoter in Intron 17

**DOI:** 10.1007/s12035-024-04293-3

**Published:** 2024-06-28

**Authors:** Rashed Rezwan Parag, Takahiro Yamamoto, Kiyotaka Saito, Dan Zhu, Liquan Yang, Erwin G. Van Meir

**Affiliations:** 1 Laboratory of Molecular Neuro-Oncology, Department of Neurosurgery, Heersink School of Medicine, University of Alabama at Birmingham (UAB), WTI 520E, 1824 6th Avenue South, Birmingham, AL 35233 USA; 2 Graduate Biomedical Sciences, University of Alabama at Birmingham (UAB), Birmingham, AL USA; 3 Present Address: Department of Neurosurgery, Kumamoto University, Kumamoto, Japan; 4 Department of Neurosurgery, Emory University School of Medicine, Atlanta, GA USA; 5 O’Neal Comprehensive Cancer Center, University of Alabama at Birmingham (UAB), Birmingham, AL USA

**Keywords:** Adhesion GPCR, ADGRB1, BAI1, Alternative promoter, Transcription variants, Protein isoforms

## Abstract

Brain-specific angiogenesis inhibitor 1 (BAI1) belongs to the adhesion G-protein-coupled receptors, which exhibit large multi-domain extracellular N termini that mediate cell–cell and cell–matrix interactions. To explore the existence of BAI1 isoforms, we queried genomic datasets for markers of active chromatin and new transcript variants in the *ADGRB1* (adhesion G-protein-coupled receptor B1) gene. Two major types of mRNAs were identified in human/mouse brain, those with a start codon in exon 2 encoding a full-length protein of a predicted size of 173.5/173.3 kDa and shorter transcripts starting from alternative exons at the intron 17/exon 18 boundary with new or exon 19 start codons, predicting two shorter isoforms of 76.9/76.4 and 70.8/70.5 kDa, respectively. Immunoblots on wild-type and *Adgrb1 exon 2*-deleted mice, reverse transcription PCR, and promoter-luciferase reporter assay confirmed that the shorter isoforms originate from an alternative promoter in intron 17. The shorter BAI1 isoforms lack most of the N terminus and are very close in structure to the truncated BAI1 isoform generated through GPS processing from the full-length receptor. The cleaved BAI1 isoform has a 19 amino acid extracellular stalk that may serve as a receptor agonist, while the alternative transcripts generate BAI1 isoforms with extracellular N termini of 5 or 60 amino acids. Further studies are warranted to compare the functions of these isoforms and examine the distinct roles they play in different tissues and cell types.

**Supplementary Information:**

The online version contains supplementary material available at 10.1007/s12035-024-04293-3.

## Introduction

Brain-specific angiogenesis inhibitor 1 (BAI1) is a member of the adhesion G protein-coupled receptors (ADGRs), a class of 33 GPCRs in humans that exhibit long multi-domain extracellular regions mediating cell–cell and cell–matrix interactions [1, 2]. Human BAI1 is a transmembrane receptor with a predicted size of 173.5 kDa and a modular structure. Sequentially from the N terminus, the extracellular region of BAI1 starts with a signal peptide for intracellular transport, an RGD (Arg-Gly-Asp) integrin-binding motif, and five thrombospondin type-1 repeats (TSRs), that can interact with CD36 on endothelial cells [3] and bind to phosphatidylserine on apoptotic cells [4] and RTN4R on neurons [5]. The TSR domain is followed by a hormone-binding domain (HBD) of an unknown ligand and a GPCR autoproteolysis-inducing (GAIN) domain that can stimulate autoproteolytic cleavage at an adjacent GPCR proteolysis site (GPS). This cleavage separates the N-terminal extracellular fragment (NTF) from the rest of the receptor, leaving a membrane-bound C-terminal fragment (CTF) with a short (~ 19 amino acid) extracellular stalk that can function as an agonist and lead to BAI1 activation [6]. The 7-helical transmembrane (7TM) domain anchors BAI1 to the cell membrane [7]. The first intracellular loop binds to the MDM2 E3-ubiquitin ligase [8], while the third loop binds to Ga_12/13_ and can activate Rho signaling [9]. Finally, the intracellular C terminus features a proline-rich region (PRR) that interacts with IRSp53, an adaptor protein that links membrane-bound small G-proteins to cytoplasmic effector proteins [10], a helical domain (HD) that can recruit ELMO/DOCK180 and activate Rac1 signaling [11], a nuclear localization sequence [12], and ends with amino acids QTEV (Gln-Thr-Glu-Val), which function as a docking site for PDZ domain-containing proteins [10] such as scaffolding protein MAGI-3, PSD-95, and Tiam1/Par3 that activates Rac1 signaling [9, 13, 14].

BAI1 was initially studied for the anti-angiogenic properties of the TSRs found in its N terminus and its overexpression can inhibit cancer growth [1, 15]. The extracellular region of BAI1 can be cleaved by MMP14, after the first TSR, to release a fragment with a predicted size of 34.7 kDa (vasculostatin-40) and autoproteolytically at the GPS site to release the NTF (also called vasculostatin-120, predicted size: 101.5 kDa), both of which have anti-angiogenic properties [3, 16]. BAI1 expression is elevated in the brain [17] but epigenetically silenced in brain tumors (gliomas and medulloblastomas) and has an anti-cancer function by trapping MDM2, which stabilizes the p53 tumor suppressor [8]. BAI1 was also reported to serve as an engulfment receptor for apoptotic cells and bacteria in macrophages [4] and promote myogenesis, which relates to muscle development and repair [18] through ELMO/Dock180/Rac1 signaling.

BAI1 is highly expressed in glial cells and neurons of the hippocampus, thalamus, amygdala, cortex, and striatum [19], and its loss leads to deficits in neurogenesis and brain function. *Adgrb1*^exon2−/−^ mice that lack full-length BAI1 expression have reduced expression of post-synaptic density 95 (PSD-95) and exhibit deficits in spatial learning and memory, and alterations in synaptic plasticity [13]. During brain development, these mice show a decrease in brain weight with reduced neuron density and increased apoptosis in the hippocampus [17]. They also show significant social behavior deficits and increased vulnerability to seizures [17].

BAI1 is located in the post-synaptic membrane of neurons, and its knockdown leads to the immature development of dendritic spines and excitatory synapse formation [14, 20]. BAI1 regulates synaptogenesis by its interaction with neuroligin-1 (NL-1), a synaptic organizer, and through the recruitment of the Par3/Tiam1 polarity complex [6, 14]. In the post-synaptic density (PSD), BAI1 promotes RhoA activation through coupling to Gα_12/13_ to maintain synaptic plasticity via microtubules’ and microfilaments’ rearrangement [9]. Also, an interaction between BAI1 and BCR (breakpoint cluster region) restricts dendritic growth via RhoA activation [20]. The extracellular domain of BAI1 is involved in promoting synaptogenesis and inhibiting dendrite and axonal growth through a high-affinity interaction between RTN4R and the third TSR domain of BAI1 [5].

BAI1 is encoded by the *ADGRB1* (adhesion G-protein-coupled receptor B1) gene located on human chromosome 8q24.3, spanning ~ 95.4 kb pairs and featuring 31 exons. Large genes can give rise to multiple protein isoforms with different functions and tissue expression profiles. Such proteomic diversity stems from a variety of mechanisms, including alternative transcription start sites driven by alternative promoters (APs) [21, 22], alternative splicing (AS) of pre-mRNAs [23], alternative polyadenylation [24], alternative translation initiation [25], and translation of non-canonical open reading frames (ORFs) [26]. In eukaryotes, these regulatory mechanisms are mainly responsible for structural and functional diversity [27]. In normal physiology, alternative promoters allow for differential expression in different cell types or tissues and are one of the dominant contributors to proteome diversity [28]. Transcriptional heterogeneity can give rise to protein isoforms with rearranged domains and altered functions [29] as well as totally different proteins with distinct functionality [30]. Alternative promoter usage and alterations in the splicing machinery can also contribute to disease formation, particularly in cancer [31, 32].

Little is known about the existence of BAI1 isoforms and the roles they play in the regulation of the above multiple biological processes, either physiologically or pathologically. Here, we investigated the different transcripts originating from the *ADGRB1* gene and resulting protein isoforms of BAI1 and evidenced new BAI1 isoforms that originate from a heretofore unknown alternative promoter in intron 17 of *ADGRB1*.

## Materials and Methods

### Cell Culture

Human glioma stem cell (GSC) lines (GSC18) and group 4 medulloblastoma (CHLA-01-MED) cell lines were cultured in a neural stem cell medium as described [33]. Human cell lines derived from glioblastoma (LN319, LN444, LN751) and SHH (ONS76 and DAOY), group 3 (D556) and WNT (UW288-1) medulloblastoma, and 293 T cells were obtained from ATCC and cultured in DMEM as described [8]. Human brain tissues were obtained from the UAB Tissue Biorepository under Institutional Review Board (IRB) approval. The cells were regularly tested for mycoplasma and their identity was verified by STR profiling.

### Mice

The full-length BAI1 knockout mouse model in which Exon-2 (containing the translation initiation codon) was replaced by homologous recombination with a promoterless *LacZ* (β-galactosidase) gene, a *neo* gene (neomycin resistance gene) followed by a stop codon and poly-A tail has been previously described [13]. Protein extracts from C57BL/6 wild type (WT), *Adgrb1*^*exon2+/-*^ (heterozygous deletion, (HET)), and *Adgrb1*^*exon2−/−*^ (homozygous deletion, knockout (KO)) mouse brain samples were prepared as described [17]. Protocols for in vivo experiments were approved by the Institutional Animal Care and Use Committee (IACUC).

### Cell Transfection and Small-Interfering (si)-RNA Knockdown

The Tet-on 3G doxycycline-inducible expression system (Takara Bio, 631,339) was used to induce the expression of full-length *ADGRB1* cDNA under the TRE3G promoter in MB (ONS76 and DAOY) and glioblastoma (LN229) cell lines according to the manufacturer’s protocol. Briefly, 293 T cells were transfected with pLVX-CMV-Tet3G (regulator vector) or pLVX-TRE3G-BAI1 (response vector) along with psPAX2/pMD2.G packaging vectors by FuGene HD (Promega, E2311) to harvest lentiviral particles. MB and glioblastoma cells were treated with lentivirus-Tet3G (pLVX-CMV-Tet3G) and selected with G418 (2 mg/ml) to generate TET3G cells. TET-3G cells were then infected with viral particles from the response vector and selected with puromycin (10 µg/ml) for Tet-On inducible BAI1 cells. The expression of BAI1 was confirmed in media containing doxycycline (Dox; 1 μg/ml). For siRNA-mediated knockdown experiments, 70–90% confluent cells were cultured in a 6-well plate and siRNA transient transfection was performed with RNAiMAX (Invitrogen, 13,778,075) as prescribed. The following siRNAs were used: siBAI1#Exon-10 (Invitrogen, AM16708, ID:4161) targeting exon 10 and siBAI1#Exon-23 (Invitrogen, 4392420, ID: s1870) targeting exon 23 of ADGRB1 (NM_001702.2) and FAM-labeled negative control, #Scramble (Invitrogen, AM4620).

### Immunoblotting

Western blots were performed on unboiled protein extracts (see Suppl. Materials and Methods) as boiling can result in BAI1 aggregates that do not migrate well through the stacking gel (Supplementary Figure S1A). Three primary antibodies targeting different epitopes of BAI1 were used: i) N-BAI1 antibody, targeting the N-terminal epitope at 103–118 amino acids (hereafter aa) [34], ii) C-BAI1 [a] antibody, targeting the C-terminal epitope at 1305-1318 aa [9] and iii) C-BAI1 [b] antibody, targeting another epitope (1537–1567aa) at the C-terminal BAI1 (ABCEPTA, AP8170a; 1:1,000). In addition, antibodies targeting pERK1/2 (Thr202/Tyr204) (Cell Signaling, #9101; 1:1000), GAPDH (Santa Cruz, sc-47724; 1:1,000), β-Actin (Santa Cruz, sc-69879; 1:1000), and fibronectin (Santa Cruz, sc-8422; 1:1,000) were also used. ImageJ 1.54d (NIH) software was used to quantify bands in immunoblots. SnapGene 3.1.4 was used for the prediction of m.w. of different BAI1 isoforms.

### RNA Isolation and Reverse Transcription PCR (RT-PCR)

Total RNA from cells and tissues was isolated using TRIzol reagent (Ambion, 15,596,018) as prescribed. The isolated RNA was diluted to 50 ng/μl. The RNA was then reverse transcribed into cDNA using iScript Reverse Transcription Supermix (Bio-Rad, 1,708,841). RT-PCR was performed using gene-specific primers (see Suppl. Materials and Methods) and Taq DNA polymerase (Qiagen, 201,203).

### Cloning and Testing of Intron 17 Promoter Region Using a Luciferase Reporter Assay

The 2210 and 1190 bp long genomic fragments in the 3ʹ region of human intron 17 were amplified by PCR. Primers are listed in the supplementary methods section. Conditions for PCR amplification were 95 °C for 2 min, followed by 35 cycles comprising denaturation at 95 °C for 30 s, annealing at 64 °C for 60 s, and extension at 68 °C for 3 min, with a final extension at 68 °C for 10 min. PCR amplified fragments were cloned into the pGL2-basic-firefly luciferase reporter plasmid using the XhoI and HindIII restriction sites. The pGL2-CMV-firefly luciferase plasmid was used as a positive control. LN229 and HEK293T cells were plated in 24-well plates at 1 × 10^5^ cells/well. At ~ 80% confluency, a total of 1 µg 2210 or 1190 intron 17 fragment-driven firefly luciferase plasmids and a transfection control plasmid (pLV-EF1-RenillaLuc plasmid) were transiently transfected into LN229 and HEK293T cells. After 48 h of transfection, luciferase activity was measured in a 20/20n Single Tube Luminometer (Turner BioSystems, 2030–000) using a Dual-Luciferase Reporter Assay System kit (Promega, E1910) as prescribed. Firefly luciferase activity was normalized to the activity of Renilla luciferase. The experiments were performed in three independent biological replicates and reactions of each sample were carried out in triplicate.

### Computational Biology Analyses

Normalized mRNA expression levels (nTPM) of *ADGRB1* in different regions of human and mouse brains were analyzed through The Human Protein Atlas (www.proteinatlas.org) [35]. To assess the exon-specific and transcripts-specific expression of *Adgrb1* in mice brains, we analyzed the bulk RNAseq dataset generated by Zhang et al. (GSE52564) [36] and further quantile normalized by McKenzie et al. [37]. Histone ChIP-seq data from the ENCODE portal (www.encodeproject.org) [38, 39] were queried for histone modification marks with the following dataset identifiers: ENCSR875PYX for H3K4me3 ChIP-seq and ENCSR581KPP for H3K27ac ChIP-seq, in dorsolateral prefrontal cortex tissue of adult human; ENCSR000CAK for H3K4me3 ChIP-seq and ENCSR000CDC for H3K27ac ChIP-seq in cerebellum tissue of adult mice. To determine chromatin accessibility, we also extracted ATAC-seq data from the cerebellum tissue of humans (dataset identifier: ENCSR802GEV) and mice (dataset identifier: ENCSR554JQP) from the ENCODE portal [38, 39]. For the visualization of ChIP-seq and ATAC-seq data, we used Integrative Genomics Viewer (IGV-V2.12.2, Broad Institute). Long-read (LR) RNA seq of breast cancer data was analyzed with Breast Cancer Long Read Transcriptome (brca-isoforms.jax.org) [40]. Further analysis of full-length transcript sequencing data of the human cortex [41] (SRA: PRJNA664117) and mouse cortex [41] (SRA: PRJNA663877) was visualized by using the UCSC genome browser. Gene promoter candidate regions were predicted with Ensembl Release 110 (useast.ensembl.org) [42]. Alternative transcription start sites were predicted through the DataBase of Transcriptional Start Sites (DBTSS; dbtss.hgc.jp) [43, 44]. Exon-specific expressions of *ADGRB1* mRNA variants were visualized by the Genotype-Tissue Expression (GTEx) database (www.gtexportal.org/home/) [45]. The median read counts per base for each exon were calculated based on a collapsed gene model where all *ADGRB1* mRNA variants are combined into a single transcript by using a heatmap.

### Statistical Analyses

GraphPad Prism 9.0 (GraphPad, La Jolla, CA, USA) was used for biostatistics. Data are expressed as mean ± SEM. One-way ANOVA with Tukey’s multiple comparisons correction was performed to identify statistical differences. *P* < 0.05 was considered statistically significant.

## Results

### Evaluation of BAI1 Protein Isoforms in Mouse and Human

**Fig. 1:**
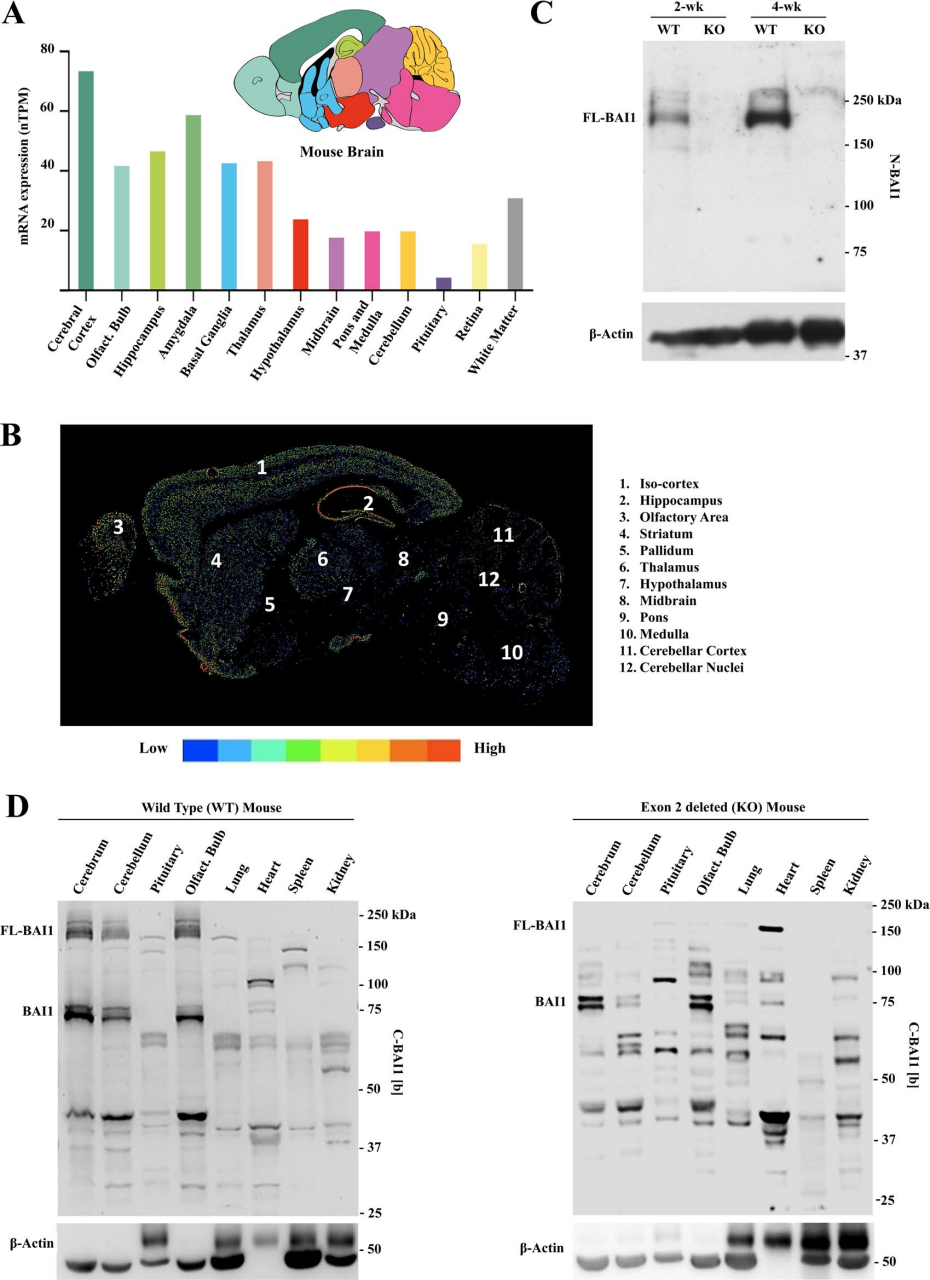
Assessment of *Adgrb1* mRNA variants and BAI1 protein isoforms in mouse tissues. **A** *Adgrb1* mRNA expression in 13 mouse brain regions. Source: The Human Protein Atlas (www.proteinatlas.org) accessed on February 18, 2024 [35]. nTPM, normalized transcript per million; olfact. bulb, olfactory bulb. **B** In situ hybridization of *Adgrb1* mRNA expression in C57BL/6 mouse brain (postnatal day 56). Anatomical regions are indicated. The color scale indicates transcript expression levels. Source: Allen mouse brain atlas (mouse.brain-map.org/experiment/show/71283681) accessed on February 18, 2024 [46]. **C** Western blot of BAI1 protein in brain extracts of wild-type (WT) and exon 2 deleted (KO) C57BL/6 mice (age: 2 weeks and 4 weeks) by using N-BAI1 antibody (epitope 103–118aa). **D** Expression of BAI1 isoforms in different organs and brain regions of WT (left panel) and exon 2 deleted (KO) C57BL/6 mice (right panel) by using C-BAI1 [b] antibody (epitope 1537–1567aa)

At first, we examined mRNA expression levels of *Adgrb1* in mice with a particular focus on the brain [35]. Analysis of 13 main brain regions evidenced the highest expression in the cerebral cortex and lowest in the pituitary (Fig. 1A). Spatial distribution and intensity of *Adgrb1* transcripts were revealed by in situ hybridization [46]. A relatively high expression of *Adgrb1* was identified in the iso-cortex, hippocampus, and olfactory bulb and low in the midbrain, pons, medulla, and cerebellum (Fig. 1B).

Next, we used western blots to examine BAI1 protein levels in brain extracts of wild-type (WT) and *Adgrb1 *^*exon 2−/−*^ mice. The latter lack full-length BAI1 expression due to deletion of exon 2, which contains the ATG start codon in full-length *Adgrb1* transcripts [13]. Probing the immunoblot membranes with an anti-N-terminal BAI1 antibody, N-BAI1 (antigen region: 103–118 aa) [34], revealed a main band at ~ 200 kDa in WT mice, which was absent in the exon 2-deleted mice as expected (Fig. 1C). Probing extracts from different brain regions and main organs with a C-terminal antibody (C-BAI1 [b]) showed predominant bands in the ~ 160–200 kDa and ~ 70–75 kDa size ranges in WT mice, with additional smaller isoforms around ~ 40 kDa (Fig. 1D, left panel). A similar analysis of BAI1 isoforms in exon 2-deleted (KO) mice showed that the ~ 160–200 kDa isoforms representing full-length ~ 200 kDa BAI1 and some of their cleavage products (~ 160 kDa) disappeared as expected (Fig. 1D, right panel). The shorter ~ 70 and 75 kDa and ~ 40 kDa BAI1 isoforms were retained in the cerebrum, cerebellum, and olfactory bulb, suggesting that they originate from new transcripts starting downstream of exon 2. Interestingly, the pituitary and heart showed new BAI1 isoforms in the *Adgrb1 *^*exon 2−/−*^ mice (Fig. 1D, and Supplementary Figure S1B), which will require future investigation. Altogether, these data suggest that short BAI1 isoforms are produced in mouse brain cells, some the result of alternative transcripts and others from proteolytic cleavage of higher molecular weight BAI1.

**Fig. 2:**
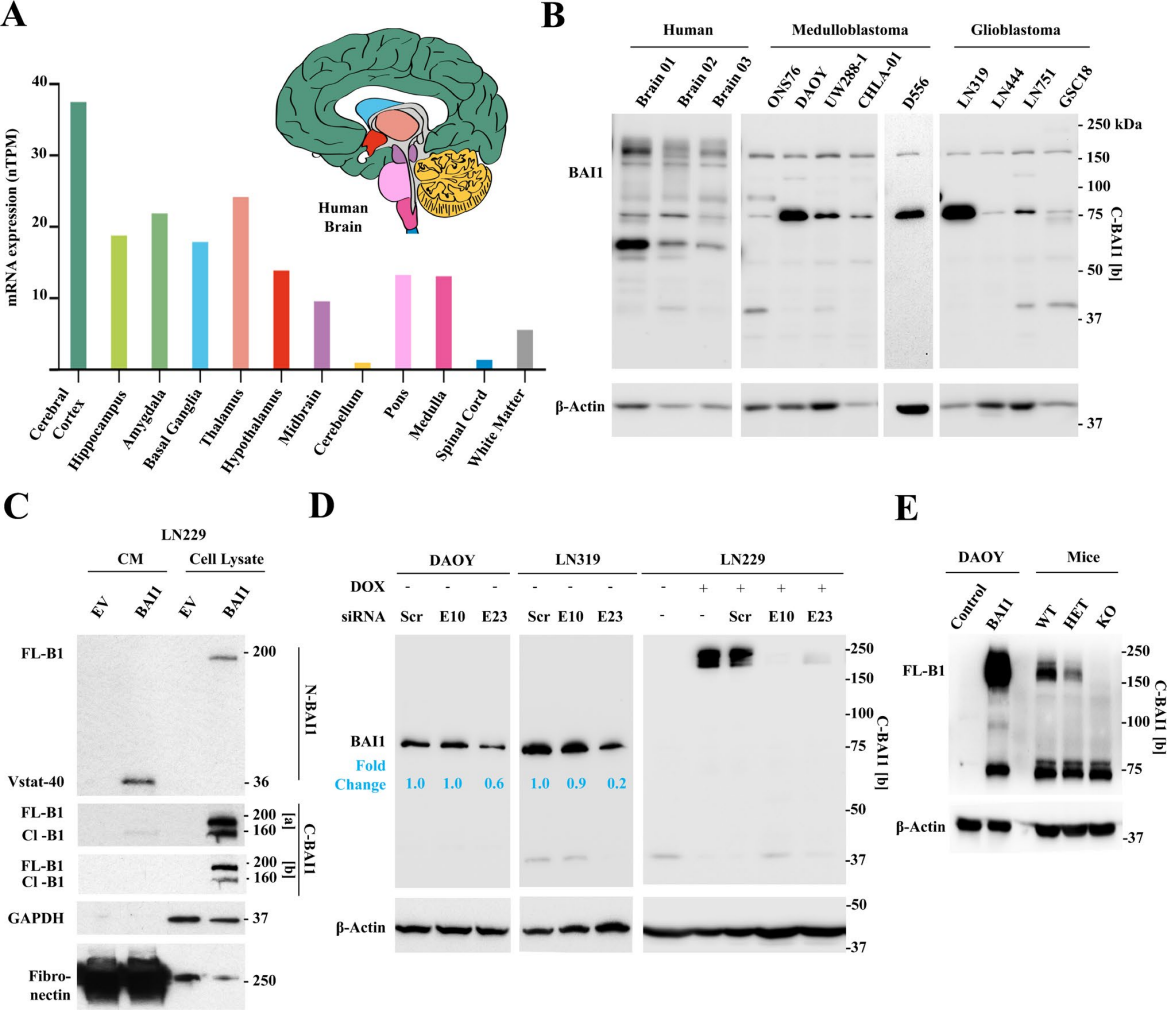
Investigation of *ADGRB1* mRNA variants and BAI1 protein isoforms in human tissues. **A** Normalized mRNA expression levels (nTPM) of *ADGRB1* in 12 regions of the human brain. Source: The Human Protein Atlas (www.proteinatlas.org) accessed on February 18, 2024 [35]. **B** Western blot analysis of BAI1 in normal human brain, human medulloblastoma (ONS76, DAOY, UW288-1, CHLA-01, and D556), and glioblastoma (LN319, LN444, LN751) cell lines and human glioma stem cells (GSC18) using a C-BAI1 [b] antibody (epitope: 1537–1567aa). **C** Western blot analysis of BAI1 isoforms present in conditioned media (CM) and cell lysates of LN229 cells transfected with empty vector (EV) or BAI1 overexpression vector (BAI1). N-BAI1 (epitope: 103–118aa) [34], C-BAI1 [a] (epitope: 1305–1318aa) [9], and C-BAI1 [b] (epitope: 1537–1567aa) antibodies were used. Note: FL-BAI1 migrates at ~ 200 kDa, while its cleavage at the N-terminal MMP14 site generates a 160 kDa truncated form and a soluble ~ 40 kDa fragment (Vasculostatin-40, Vstat-40). FL-B1 means full-length BAI1, Cl-B1 means cleaved BAI1. **D** BAI1 protein level after siRNA-mediated knockdown of *ADGRB1* in DAOY and LN319 cell lines. SiRNAs targeting exon 10 and exon 23 of *ADGRB1* (NM_001702.2) were used. Scramble siRNA was used as a negative control. LN229 cells expressing tet-on FL-BAI1 cDNA (1 mg/ml doxycycline for 48 h) were used as a positive control for the efficacy of siRNAs. Relative fold changes in ~ 75 kDa BAI1 levels are indicated (normalized to β-Actin). C-BAI1 [b] antibody was used. **E** Western blot comparing BAI1 isoforms between medulloblastoma cells and mice brain (WT, HET, and KO C57BL/6 mice). FL-BAI1 (~ 200 kDa) and autoproteolytically cleaved BAI1 (~ 72 kDa) are detected in DAOY MB cells transfected with BAI1 cDNA. Isoforms of ~ 72 and 75 kDa are seen in WT and KO mice cells, suggesting they both derive from alternative transcripts. Anti-C-terminal BAI1 (C-BAI1 [b]) was used

As in mice, analysis of *ADGRB1* expression in the human brain showed the highest mRNA levels in the cerebral cortex, and lowest in the cerebellum and spinal cord (Fig. 2A). Examination of BAI1 protein expression with an anti-C-terminal antibody (C-BAI1 [b]) in normal human brain tissue revealed major bands of ~ 150–200 kDa, representing isoforms of full-length BAI1 (FL-BAI1) with variable post-translational modifications (Fig. 2B). Transfection of full-length BAI1 cDNA in glioma cells showed that the ~ 150–160 kDa band represents an isoform with a shortened N terminus (Fig. 2C), likely due to MMP14 cleavage of FL-BAI1 [16]. As expected, FL-BAI1 expression was strongly reduced in adult glioblastoma and pediatric medulloblastoma cells compared to normal brains (Fig. 2B), as FL-BAI1 is silenced in these cancers [8, 34, 47]. Additional smaller bands were observed in the normal human brain around 75 kDa, and their expression was maintained or even increased in the tumor cells (Fig. 2B). To explore their origin, we used RNA interferenc-mediated silencing. siRNAs targeting *ADGRB1* exon 23, but not exon 10, reduced the expression of ~ 75 kDa BAI1 isoforms in medulloblastoma (DAOY) and glioblastoma (LN319) cell lines (Fig. 2D, left and middle panels). Both siRNAs are functional as they led to the reduction of exogenously overexpressed FL-BAI1 in LN229 glioblastoma cells (Fig. 2D, right panel). These findings indicate that the short isoforms are derived from alternative *ADGRB1* gene transcripts downstream of exon 10 and are not the result of non-specific antibody binding. To compare this band to the one resulting from autoproteolytic cleavage of FL-BAI1 at the GPS site, we explored their relative sizes by transfecting FL-BAI1 cDNA in medulloblastoma cells (Fig. 2E and Supplementary Figure S1C). Transfected cells displayed a ~ 200 kDa band representing FL-BAI1 and a smaller ~ 72 kDa band likely representing the result of autoproteolysis (Fig. 2E). These human isoforms of BAI1 are very similar in size to those observed in mice (Fig. 2E).

Overall, these data show strong similarities between human and mouse brain BAI1 isoforms, with the expression of shorter ~ 70–75 kDa isoforms which can originate from autoproteolysis of FL-BAI1 or derive from alternative transcripts.

### Cell-Type Specific Expression of Adgrb1 Exons and Transcripts in the Mouse Brain

**Fig. 3:**
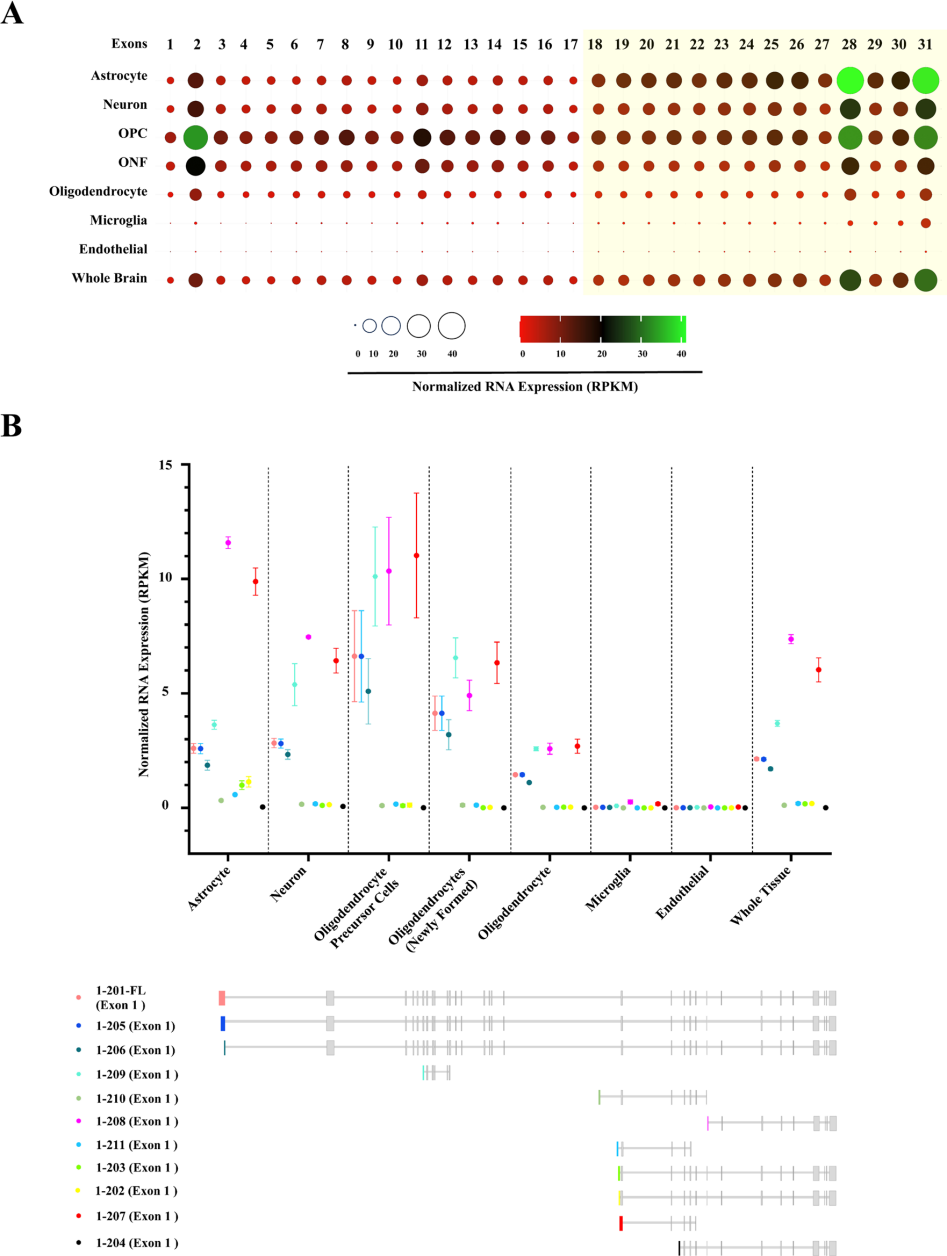
Cell-type-specific exonic and transcriptomic variation of *Adgrb1* in mice brain. **A** Exon-specific normalized mRNA expression of *Adgrb1* in different cellular populations of the mouse brain (mouse reference genome: mm10). Circle sizes and tile colors in the heat map represent the Read Per Kilobase per Million mapped reads (RPKM) values for each exon of *Adgrb1.* OPC, oligodendrocyte precursor cells; ONF, oligodendrocytes (newly formed). The figure was generated by using SRplot [48]. **B** Cell-type-specific transcriptomic analysis of *Adgrb1* in mice brain (mm10). Normalized mRNA expression level (RPKM values) of eleven (11) known *Adgrb1* transcripts of mice (transcript source: Ensembl genome browser, release 110). Each transcript has a unique first exon indicated by a distinct color. Values indicate the arithmetic mean and standard error of the mean of the RPKM

Distinct brain cell populations display distinct transcriptional patterns that govern unique cell-specific functions. To probe for the *Adgrb1* expression profile in different mouse brain cell populations, we queried exon-level usage and transcript-specific expression of *Adgrb1* in bulk RNAseq datasets [36, 37]. We identified a notable increase in the expression of Exons 18–31 of *Adgrb1* in astrocytes, neurons, and the whole brain, compared to Exons 1–17 (Fig. 3A). To confirm transcriptomic variation of *Adgrb1*, we further analyzed the cell-type specific expression level of 11 known *Adgrb1* transcripts identified in Ensembl genome browser Release 110 [42]. Remarkably, two short transcripts, *Adgrb1*-207 (ENSMUST00000187599.2) and *Adgrb1*-208 (ENSMUST00000187639.2), exhibited the highest expression levels across all brain cell types (Fig. 3B). These findings confirm the presence of short *Adgrb1* transcripts and the existence of alternative transcription start sites near exon 18 of full-length *Adgrb1*.

### Analysis of the Mouse Adgrb1 Gene Transcriptional Heterogeneity

**Fig. 4:**
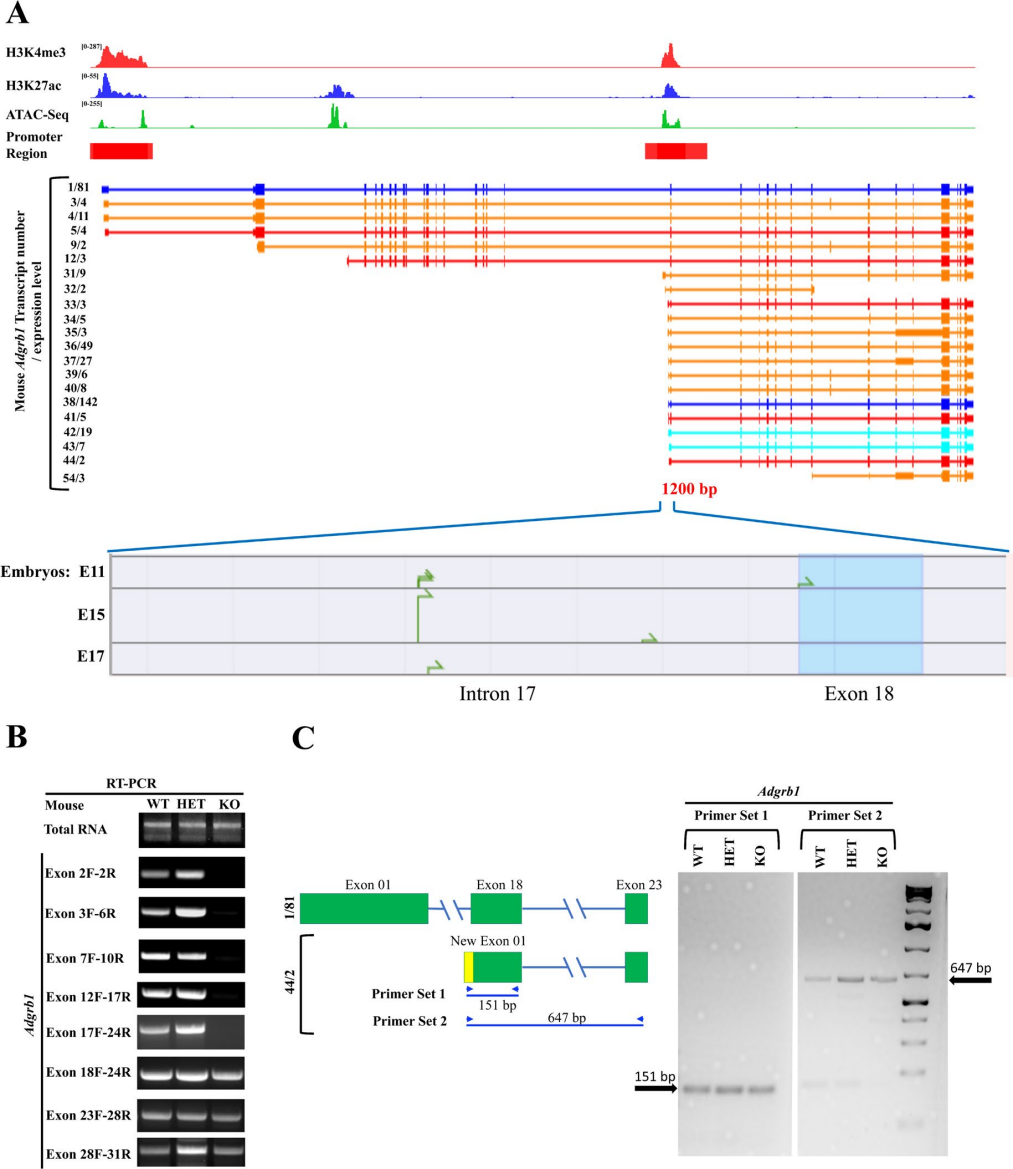
Transcriptional diversity of the mouse *Adgrb1* gene. **A** Top: Genome browser tracks of H3K4me3 and H3K27ac ChIP-seq data (cerebellum tissue), ATAC-seq data (cerebellum tissue), Iso-seq data, and TSS-seq data as well as predicted promoter regions near exons 1 and 18 from Ensembl genome browser (red rectangles) at the mouse *Adgrb1* locus (mm10) in adult mouse from C57BL/6 background. Middle: *Adgrb1* transcript variants identified in mouse cerebral cortex by Iso-seq. The transcript number/number of independent full-length reads supporting this variant structure are indicated on the left. Transcript categories: blue = FSM; cyan = ISM; red = NNC; orange = NIC [41]. Bottom: Transcription start sites predicted by TSS-seq in a ~ 1200 bp region at the intron17/exon 18 boundary in mouse embryo brains at days E11, E15, and E17. **B** Reverse transcription-polymerase chain reaction (RT-PCR) analysis of exon usage in *Adgrb1* transcripts in the brain of wild-type (WT), heterozygous (HET), and exon 2 deleted (KO) mice using exon-specific forward (F) and reverse (R) primers. **C** RT-PCR analysis showing the expression of novel *Adgrb1* transcript variant (44/2) from the alternate promoter in intron 17 in mouse brain. Left: Exon structure and primer design of transcript 44/2 are shown. Yellow, start of new exon 1 from intron 17 start site. Green, exons are present in full-length transcript. Right: agarose gel showing RT-PCR products in WT, HET, and KO mice

To identify alternative promoters and potential transcriptional start sites across *Adgrb1*, we queried ChIP-seq results mapping the distribution of histone modification marks across the mouse gene. We further extended this data with ATAC-seq, which identifies regions of open chromatin [49]. Two main transcriptionally active open chromatin regions were revealed by ATAC-seq and display strong enrichment of H3K4me3 and H3K27ac, two marks of transcriptionally active promoter regions [50]. One is located near exon 1 and the other straddles the intron 17/exon 18 boundary, and both are predicted to be gene promoters by the Ensembl genome browser (Fig. 4A, top panel). DBTSS database analysis identified some putative TSS in a 1000 bp region near exon 18 in the mouse embryo (Fig. 4A, bottom panel). A third region upstream of exon 3 also revealed open chromatin but with weak H3K4me3 marks, perhaps representing a weaker promoter as it matches the start of transcript 12/3.

To examine how these open/active chromatin regions direct the transcriptional heterogeneity of mouse *Adgrb1*, we aligned them with transcripts identified by long-read isoform sequencing (Iso-seq) of mouse cerebral cortex [41] (Fig. 4A). We identified a total of 21 *Adgrb1* transcripts (Fig. 4A, middle panel): 5 transcribed from exons 1 or 2 and 14 from the 3ʹ end of intron 17, matching the two main open/active chromatin areas. Ten of the latter transcripts have new exons in intron 17 that splice to exon 18. Three (42/19, 43/7, and 44/2) have new first exons that are a 5ʹ extension of exon 18. All of these use a start codon in exon 19. The last transcript (41/5) has an alternative new exon in intron 17 that splices to the middle of exon 18 and uses a new ATG. Careful examination of the reading frame of each newly spliced transcript suggests that most of the mRNAs transcribed from the 3′ end of intron 17 have a start codon in exon 19 (including transcripts 38/142, 42/19) and are predicted to generate a mBAI1 (mouse BAI1) isoform of 642 amino acids (aa) (predicted size: 70.5 kDa). In contrast, transcript 41/5 is translated from a new ATG and generates a 700aa mBAI1 isoform (predicted size: 76.4 kDa) (see details in Supplementary Table S1).

To independently validate the existence of these variant mRNA transcripts in the mouse brain, we used reverse transcriptase PCR on wild-type and *Adgrb1*^*exon2−/−*^ mice [13]. RT-PCR with a series of primers spanning exons 2 to 31 failed to detect any transcripts between exons 2 and 17 in exon 2-deficient mice (Fig. 4B). However, clear mRNA expression was detected from exons 18 to 31, supporting the existence of transcripts initiated from intron 17. To validate the existence of specific transcripts, we used RT-PCR with forward primers starting in the newly predicted exons in intron 17 (serving as new exon 1) and reverse primers in exons 18 or 23. We generated a primer set for a new exon 1 that can detect putative transcripts 42/19, 43/7, and 44/2 and were successful in amplifying cDNA in WT and *Adgrb1*^*exon2−/−*^ mice (Fig. 4C). We were unable to confirm transcript 41/5 by RT-PCR in the mouse brain, likely due to the high GC content (89%) in its alternative first exon and difficulty in generating unique primers differentiating the alternative exons, so other approaches to confirm the remaining transcripts are warranted.

In conclusion, our data evidence the presence of an alternative promoter in the 3ʹ region of intron 17 of the *Adgrb1* gene. This mouse promoter can initiate transcription of multiple transcripts predicted to encode various mBAI1 proteins, ranging from 642 to 700aa with predicted sizes of 70.5–76.4 kDa, likely corresponding to the ~ 70 and ~ 75 kDa bands detected by immunoblotting.

### Analysis of the Human ADGRB1 Gene Transcriptional Diversity

**Fig. 5:**
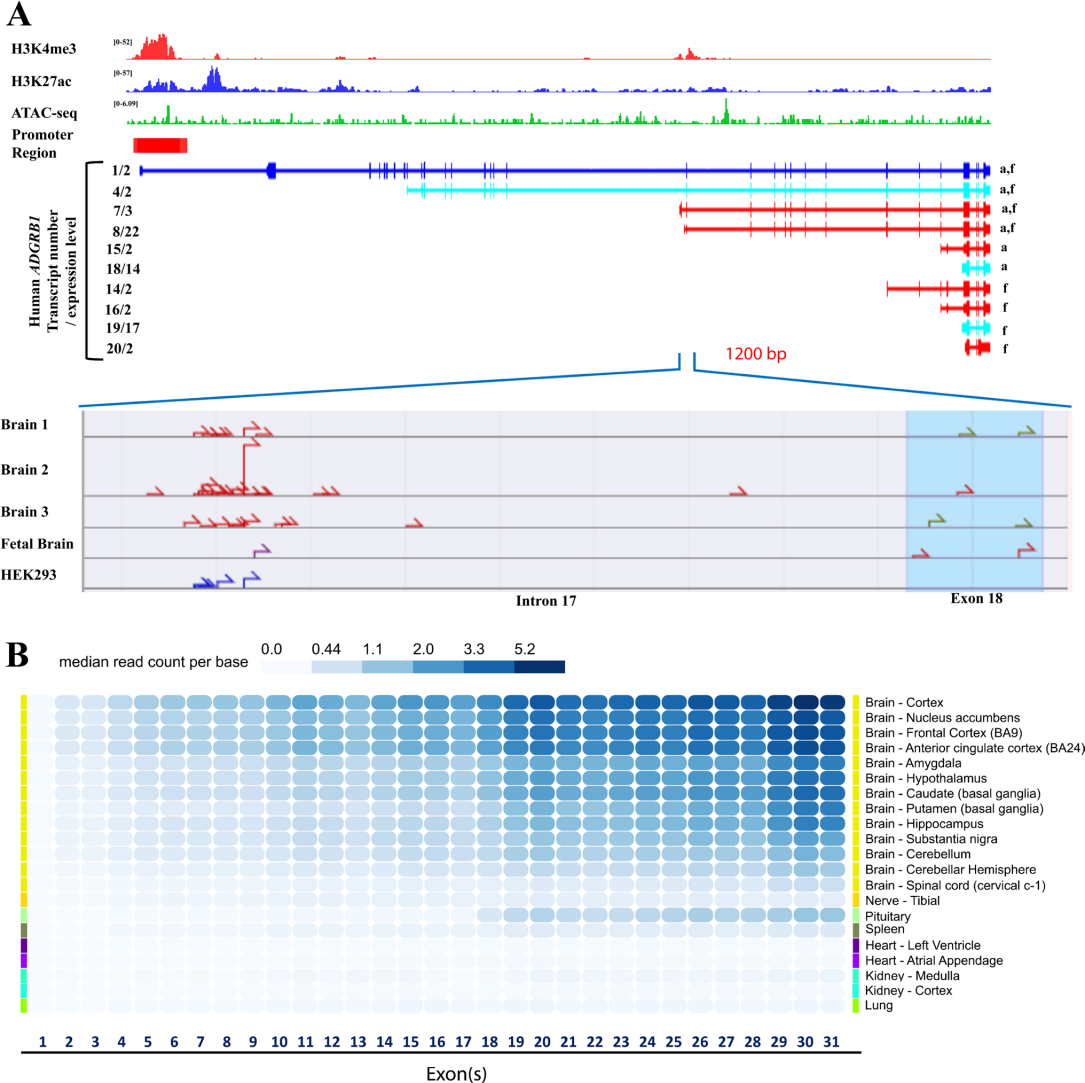
Transcriptional diversity of the human *ADGRB1* gene. **A** Top: Genome browser tracks of H3K4me3 and H3K27ac ChIP-seq data (dorsolateral prefrontal cortex), ATAC-seq data (cerebellum tissue), Iso-seq data, and TSS-seq data as well as a predicted promoter region from Ensembl genome browser (red rectangle) at the human *ADGRB1* locus (hg38). Middle: *ADGRB1* transcript variants identified in the human cerebral cortex by long-read isoform sequencing (Iso-seq). The isoform number/number of independent full-length reads supporting this isoform structure are indicated on the left. a, adult; f, fetal. Colors indicate the classification of transcript categories (blue = FSM; cyan = ISM; red = NNC; orange = NIC) [41]. Bottom: Transcription start sites predicted by TSS-seq in a ~ 1200 bp region at the intron17/exon 18 boundary in adult and fetal brains and in the HEK293 cell line. **B** Heatmap showing median read counts per base for each exon of *ADGRB1* in different brain regions and other tissues from the GTEx database. Gene-level expression is calculated based on a collapsed gene model combining all *ADGRB1* mRNA variants into a single transcript [45]

In the dorsolateral prefrontal cortex of the human brain, ChIP-seq data revealed strong enrichment of H3K4me3 and H3K27ac, two marks of transcriptionally active promoter regions [50], near exons 1 and 18, with minor peaks in introns 1 and 2 (Fig. 5A, top panel). ATAC-seq peaks were also found to overlap with those regions (Fig. 5A, top panel). Ensembl identified a candidate promoter region encompassing exon 1 and part of intron 1 (Fig. 5A, top panel, red rectangle).

Iso-seq analysis in the cerebral cortex of adult and fetal human brains [41] revealed a total of 10 mRNA variants of human *ADGRB1* (Fig. 5A, middle panel). Two (2) of the novel mRNAs (7/3, 8/22) have new alternative first exons starting in the 3ʹ end of intron 17. The alternative first exon of transcript 7/3 has a new start codon (ATG) which is in-frame with exon 18 codons of *ADGRB1* and can be translated to generate a 699aa hBAI1 (human BAI1) isoform (predicted size: 76.9 kDa). The other transcript (8/22) with an alternative first exon in intron 17 splices to exon 18, but uses a translation start codon in exon 19, forming a 644aa long hBAI1 isoform (predicted size: 70.8 kDa) (see details in Supplementary Table S2). Shorter transcripts that do not associate with an H3K4me3 mark were also observed: transcript 14/2 starts from the 5ʹ end of exon 25, has an alternative new exon in intron 27, and encodes 449aa (predicted size: 49 kDa), and 5 shorter transcripts start near exons 27 (15/2, 16/2) or 28 (18/14, 19/27, and 20/2) and will need future study. Long-read (LR) RNA seq of breast tumors also evidenced transcripts originating in intron 17 (Supplementary Figure S2), showing this is not unique to the brain [40].

To further interrogate the region near exon 18 for potential transcription start sites (TSS), we queried the DBTSS database [43, 44]. Multiple candidate TSS were identified ~ 1000 bp upstream of exon 18 in the adult and fetal normal human brain and in the HEK293 cell line (Fig. 5A, bottom panel).

In addition, to examine whether the longer and shorter *ADGRB1* transcripts lead to different exon usage in different tissues, we queried the Genotype-Tissue Expression database [45] of exon-specific reads in a collapsed gene model (Fig. 5B). This evidenced higher median read counts per base after exon 18 in the brain and pituitary, further supporting the existence of transcripts starting around exon 18 in human *ADGRB1*. It also evidences denser reads in exons 29–31, possibly resulting from the short 3ʹ transcripts (18/14, 19/17, 20/2).

In combination, these analyses support the existence of an alternative promoter region at the intron 17/ exon 18 boundary in the human brain, which is predicted to generate several transcripts that would encode hBAI1 isoforms of 644 and 699 amino acids (predicted sizes: 70.8 and 76.9 kDa, respectively), which may correspond to the ~ 70 and ~ 75 kDa bands observed by western blotting.

### Functional Testing of Alternative Promoter Activity in Human Cells

**Fig. 6:**
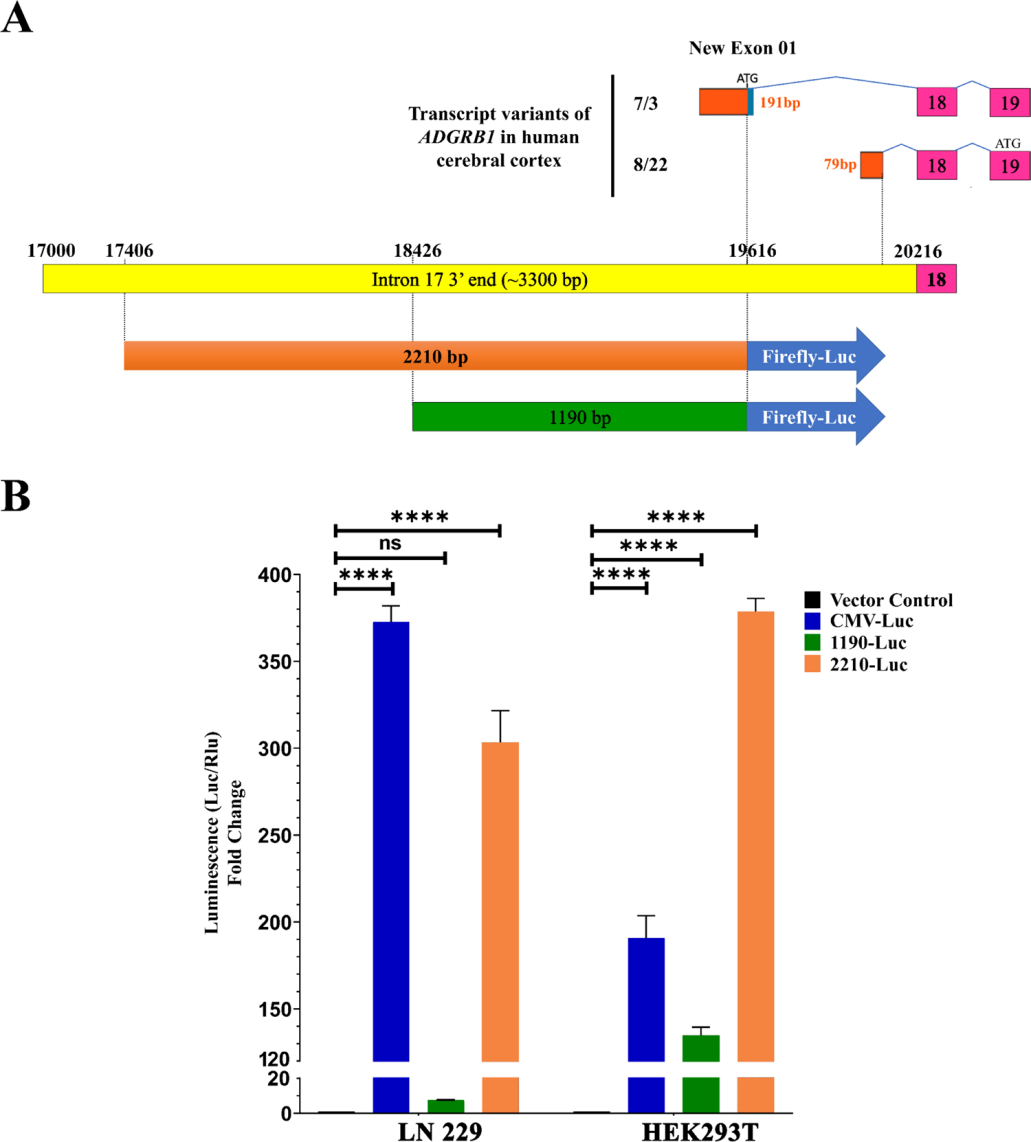
Functional evaluation of intron 17 alternative promoter activity in human cells. **A** New exon 1 of transcript *ADGRB1* variants starting at the 3ʹ end of intron 17 identified in the human cerebral cortex (orange rectangles). Length of the new exon 1, splicing to exon 18, and start codons (ATG) are indicated. The variants use the same open reading frame (ORF) as the full-length *ADGRB1*. **B** Two regions of interest (1190 bp and 2210 bp long) upstream of the new exons shown in (A) were tested for putative promoter activity in firefly luciferase reporter constructs and transiently transfected in LN229 and HEK293T cells with an EF1-renilla luciferase construct as an internal control. The fold change of the normalized firefly over  renilla luciferase activity is shown. A CMV-Luc reporter was used as a positive control (cell extracts were diluted 2000 fold). Data are presented as mean ± SEM. ^∗∗∗∗^*P* < 0.0001 (one-way ANOVA with Tukey’s multiple comparisons correction). Luc, firefly luciferase and Rlu, renilla luciferase

To functionally evaluate the presence of an alternative promoter at the 3ʹ end of intron 17 in human *ADGRB1*, we sub-cloned two overlapping genomic fragments containing the new exon region and 1–2 Kb upstream sequence (1190 and 2210 bp) (Fig. 6A) into a luciferase expression vector lacking promoter and enhancer regions. Transient transfection in cells expressing neuronal (HEK 293T) [51] or glial (LN229 glioblastoma) lineage markers revealed that the longer fragment elicited robust transcriptional activation of luciferase in both cell lines, while the shorter one showed modest activation in HEK 293T cells and none in LN229 cells (Fig. 6B). These results demonstrate that the 2210 bp distal region of intron 17 can serve as a gene promoter and contains critical regions for transcriptional activation lacking in the shorter construct.

### The Alternative Promoter in Intron 17 Leads to the Formation of ~ 70–75 kDa BAI1 Isoforms

**Fig. 7:**
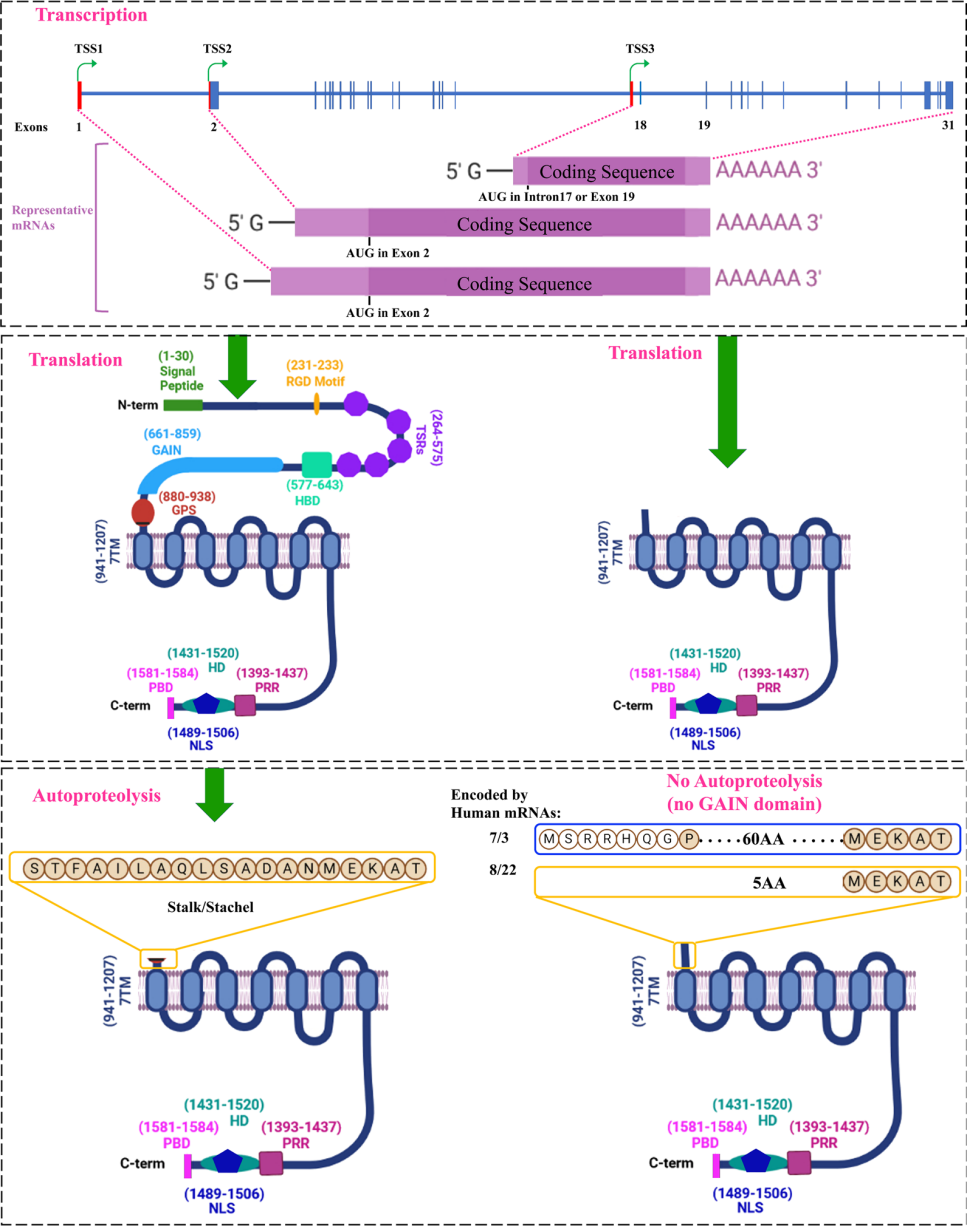
Structure of BAI1 isoforms generated from core and alternative *ADGRB1* promoters. Top panel: Schematic showing the three main transcriptional start site regions (TSS1,2,3) in the *ADGRB1* gene (exons are shown with blue rectangles). Transcription from the 5ʹ core promoter near exon 1 generates two mRNAs that start in exons 1 and 2, respectively. They differ in the size of their 5ʹ UTR regions (red rectangles) but use the same start codon in exon 2. Several transcripts are generated from the alternative promoter at the 3ʹ end of intron 17 with variable 5ʹ UTR regions and translation start codons either in the new alternate exons 1 in intron 17 or an in-frame ATG in exon 19 (see detail in Fig. 7 and Supplementary figure S2). Middle panel: Left, translation from full-size mRNAs gives rise to FL-BAI1 proteins (1584 amino acids; predicted size of 173.5 kDa) in humans. Locations of functional domains described in the introduction are indicated. Right, translation from shorter transcripts originating from an alternative promoter in intron 17 generates hBAI1 isoforms lacking most of the N-terminal region. Those using a start codon in the new exon 1 have a predicted size of 76.9 kDa, while those using the ATG in exon 19 are 70.8 kDa. These predicted isoform sizes are calculated for transcripts that share the same exons 19–31 as the full-size transcript. Bottom panel: Left, GAIN domain induced autoproteolysis at the GPS of FL-hBAI1 in the endoplasmic reticulum cleaves between leucine (L-926) and serine (S-927) and leaves a membrane-associated N-terminal truncated BAI1 with a remaining stalk of 19 amino acids (aa) [6] that can serve as an agonist (also called “stinger” or “Stachel” in German) to activate the receptor. Right, the hBAI1 isoforms generated from the alternative promoter transcripts likely do not undergo autoproteolysis as they lack the GAIN domain. The transcripts translated from the ATG in exon 19 generate a hBAI1 isoform with only 5aa outside of the cell membrane. On the contrary, those translated from an ATG in the new exon 1 in intron 17, have 60aa N termini. Amino acids (aa) compositions of N termini are shown

As proteolytic fragments of full-size BAI1 protein and the shorter BAI1s generated from the heretofore cryptic promoter in intron 17 appear similar in size (~ 70–77 kDa), we wanted to gain a finer understanding of structural differences at their N termini. FL-BAI1 human mRNAs have 31 exons and translation starts from exon-2 (Fig. 7, top panel), generating a predicted protein of 1584 amino acids (173.5 kDa), which migrates on western blots at ~ 160–200 kDa as it can undergo post-translational modifications and cleavage at the MMP14 site (Fig. 2). FL-BAI1 has a signal peptide, RGD motif, TSRs, HBD, and GAIN domain upstream of the GPS region (Fig. 7, middle-left panel). Autoproteolysis at the GPS region results in a truncated BAI1 membrane receptor of 658 amino acids (predicted size of 72.2 kDa) with a 19aa long N-terminal stalk [6], a 7TM domain, and a C-terminal region (Fig. 7, bottom-left panel). The cleaved extracellular N-terminal fragment (NTF) remains non-covalently associated with the 7TM domain of the membrane-associated C-terminal fragment (CTF) till an unknown stimulus can detach it from the receptor and lead to receptor activation by a conformational change, possibly triggered by the remaining stalk acting as a cryptic agonist [52–54].

Two short BAI1 isoforms are encoded by human transcripts generated from the intron 17 promoter. The shortest one in humans is 644aa long (theoretical size of 70.9 kDa) and does not undergo additional autoproteolysis as it lacks the GPS site. It starts with MEKAT at its N terminus, and the remaining 7TM and intracellular motifs are the same as for FL-BAI1 (Fig. 7, bottom-right panel). This shorter isoform lacks a signal peptide and cannot form a peptide agonist (“Stachel”), which may affect its cellular localization and signaling function. The second one is 699aa long and is encoded by three of the novel human mRNAs (7/3 in cerebral cortex and PB.34262.1 and 62.3 in breast tumors) that have a new start codon (ATG) in their alternative first exon that is in-frame with exon 18 codons of full-length *ADGRB1.* The predicted size of this isoform is 76.9 kDa, and it features a 60aa long N terminus (Fig. 7 and Supplementary Figure S2) with 7 novel amino acids (white circle in Fig. 7 and Supplementary Figure S2) that are absent in FL-BAI1. It has a GPS site but lacks the GAIN domain to induce autoproteolysis.

Altogether, our findings establish the presence of an alternative promoter at the 3ʹ end of intron-17 of both human and mouse *ADGRB1/Adgrb1* genes, and transcription and translation from this alternative promoter lead to the formation of two shorter BAI1s with distinct 5 and 60aa N termini in human.

## Discussion

Class B human GPCRs are comprised of 33 ADGR cell surface receptors with very long N termini that enable interactions with neighboring cells and the extracellular environment [55]. While all ADGRs share a GAIN domain and GPS, their N termini are highly variable and harbor different structural domains organized in a modular fashion, allowing them to interact with a variety of binding partners. Hence, the extracellular domains of ADGRs can mediate individual interactions with specific binding partners to carry out distinct functions or serve as a scaffold for the assembly of multiple partner proteins into a complex. ADGR receptors are encoded by large genes that can express a variety of transcripts encoding multiple protein isoforms, leading to variation in the functional domains they carry, thus impacting overall protein function [23]. A comprehensive understanding of ADGR isoforms made in different tissue types is important to unravel their function in physiology and disease.

The purpose of this study was to explore the proteomic diversity of BAI1 and start to catalog the different protein isoforms observed by immunoblotting and decipher their molecular origin. Full-length BAI1 (1584 amino acids; predicted size of 173.5 kDa) migrates at an apparent size of ~ 160–200 kDa on immunoblots. Identifying individual ADGR isoforms on Western blots can be challenging due to the extensive post-translational modifications that can occur, particularly glycosylation. Also, excessive loading of SDS on the helical hydrophobic 7-transmembrane segments can create shifts between expected and observed protein sizes [56].

Further size variation can stem from proteolytic cleavage, and prior studies have demonstrated that BAI1 can undergo two main proteolytic events [16, 57]. Cleavage between TSR1 and TSR2 by cancer-associated protease matrix metalloproteinase 14 (MMP14) yields an anti-angiogenic N-terminal fragment (Vasculostatin-40; predicted size of 34.7/35.0 kDa for human/mouse) which migrates at ~ 37–40 kDa and a remaining truncated membrane receptor (predicted size of 138.8 kDa), which migrates at ~ 160 kDa. Autoproteolytic cleavage at the GPS site generates an anti-angiogenic and anti-tumorigenic extracellular NTF (Vasculostatin-120; predicted size of 101.3/101.5 kDa for human/mouse) that migrates at ~ 120 kDa and the remainder membrane-bound CTF containing a “Stachel” peptide, 7TM domain, and C-terminal domain (predicted size of 72.2/71.8 kDa in human/mouse) that migrates at ~ 75 kDa. Thus, through two proteolytic cleavage events, five different isoforms of BAI1 can be generated: three that act at the cell surface and two in the cell environment where they might engage different targets as they will diffuse differentially due to their size and domain adhesiveness. They can be easily detected upon cDNA transfection in BAI1 silent cells using N- and C-terminal antibodies in conditioned media and cell extracts. Engineered point mutations can help define the cleavage site location and the role of surrounding amino acids. For example, we previously showed that an S927A substitution abrogated, while a phospho-mimetic S927D substitution increased the cleavage of BAI1 in glioma cells [16, 57].

An initial immunoblotting survey of the brain and other organ extracts revealed much more complexity and suggested that further isoforms are present in different tissues. The brain showed the expected full-length BAI1 at ~ 160–200 kDa and major isoforms at ~ 70–75 kDa and ~ 60 kDa (in humans only) and smaller ones at ~ 35–45 kDa. Weaker bands were also observed, and some might be non-specific signals, while others are likely different isoforms expressed at higher levels in other tissues. To distinguish isoforms resulting from proteolytic cleavage of full-length BAI1 versus those generated from potential alternative promoters we also examined the organs of mice genetically engineered with a deletion of exon 2 where the translational start site of the full-length BAI1 is located. These data evidenced that major brain isoforms of ~ 70–75 kDa and ~ 35–45 kDa were still present, showing they did not originate from proteolytic cleavage of FL-BAI1, complicating the interpretation of bands on Western blots. Unexpectedly, we also found isoforms that were only expressed in exon 2-deleted mice in the cerebrum (~ 60–65 kDa), pituitary (~ 100 kDa), and heart (~ 160 kDa and ~ 65 kDa). These may derive from other cryptic promoters and/or splicing events that are inactive in WT mice and induced upon FL-BAI1 loss, suggesting a potential regulatory network.

To further explore the origin of the BAI1 brain isoforms and determine the potential location of alternative transcription start sites and alternative splicing events, we queried long-read RNAseq data and overlayed it with information on active chromatin on the mouse and human *ADGRB1* genes. These analyses support the existence of an alternative promoter region (~ 2.5 kb) at the intron 17/exon 18 boundary and transcription start site prediction software and RNAseq reads support the existence of a cluster of transcription start sites in that region, yielding a series of mRNAs starting with new untranslated alternative exons (5’ UTRs) that splice with exon 18. To independently validate promoter activity of this intronic region, we confirmed the existence of one of the mouse transcripts by reverse transcription on exon 2-deficient mouse brain and showed that a genomic fragment of 2.2 kb just upstream of human exon 18 was sufficient to direct transcription of a reporter gene in cells expressing both glial and neuronal lineage markers. Further work is warranted to define which transcription factors are essential to activate this promoter region in different brain regions and other tissues.

Examination of the splicing pattern of the three human transcripts starting in intron 17 showed that they splice to exon 18 and retain exons 19–31 (Table S1 and S2). Other shorter transcripts starting with exons 25, 27, or 29 were also present. Some of these transcripts undergo alternative splicing with the addition of a new exon in intron 27 that adds 45aa (4.6 kDa) and will generate further diversity. In mice, 14 transcripts start from new exons in intron 17, some of which carry a new exon in intron 24, or exon rearrangements downstream of exon 26. The short transcript variants starting with exons 25–29 were not found in mice. Whether this represents a biological difference between the two species or results from the different techniques used in transcript mapping awaits further clarification. The abundance and functions of these BAI1 isoforms in diverse cells and tissue types will require further study.

The structures of the two human BAI1 isoforms produced from the alternative promoter in intron 17 are very similar to that of BAI1 resulting from proteolytic cleavage at the GPS site (Fig. 7) with slight variation in the size of the small N-terminal extracellular stalk. Autocatalytic proteolysis of FL-BAI1 induced by the GAIN domain leaves a 19aa stub that is part of a cryptic peptide (STFAILAQLSADANMEKAT) that can serve as an agonist to activate the receptor [6]. One short isoform of hBAI1 (predicted size: 70.8 kDa) is translated from an in-frame ATG in exon 19 and has a minimal N terminus (MEKAT) and is expected to lack agonistic autoactivation capability. The second type of short isoform (predicted size: 76.9 kDa) is translated from a start codon in intron-17 that is in frame with exon-18 reading frame (Fig. 7 and Supplementary Figure S2) yielding a BAI1 isoform with a 60aa N terminus. This isoform retains the GPS site, but whether it can undergo autoproteolysis is uncertain as it lacks the GAIN domain. It is tempting to speculate as to the function of these new isoforms. They might lack the receptor activity that is dependent upon the conformational change induced by the cryptic agonist but may retain other functions such as the ability to serve as a docking station for PDZ-containing proteins. Since they have a full 7-TM, they can likely bind the cleaved extracellular NTF of FL-BAI1 (Vstat120), which can repress the CTF through non-covalent association [57–59]. In situations where both isoforms are co-expressed, the shorter BAI1 might regulate the activity of FL-BAI1 by serving as a sink for Vstat-120 and thereby limiting the release of this anti-angiogenic molecule in the extracellular milieu. As cleaved CTFs can associate with NTFs released from other ADGRs [59], one may even speculate that shorter BAI1 isoforms may trap NTFs of BAI2, BAI3, or other ADGRs and in this way activate them. Such heterotypic NTF-CTF associations may also have new signaling functions that await further discovery. At first, it will be important to examine whether the new ~ 70–75 kDa BAI1 isoforms are expressed at the cell surface as they lack a signal peptide. This may not be required, as a prior study showed that a BAI1 deletion mutant lacking the entire N terminus including the “Stachel” trafficked to the plasma membrane and exhibited robust signaling activity [54]. In this context, it is useful to mention that the BAI1 C terminus harbors a domain (1489–1506aa) predicted to function as a nuclear localization sequence (NLS) [12]. It will be of particular interest to determine whether any of the newly identified BAI1 isoforms carrying this NLS preferentially traffic to the nucleus where they might exert novel functions.

Finally, this study further highlights the importance of fully characterizing the impact of gene knockdown/knockout strategies in ADGR research, so as to properly understand their biological impact. For example, our prior studies have found that *Adgrb1 *^*exon2−/−*^ mice have decreased expression of post-synaptic protein 95 (PSD95) expression, a scaffolding protein important for dendritic spines/synapse formation, and exhibit severe deficits in synaptic plasticity and hippocampus-dependent spatial learning and memory [13]. We can now conclude that these functions are dependent upon the expression of full-length BAI1 and are not rescued by the shorter isoforms. Yet, the dendritic arborization and spine density of these mice appeared similar to wt mice, contrasting with other studies targeting BAI1 acutely in neurons with shRNAs where clear spine deficits were observed [60]. While potential reasons underlying these divergent observations have already been proposed [60], this study suggests that the contribution of shorter BAI1 isoforms to dendritic spine maintenance may also be worth considering. Our data show that the expression of several isoforms is permanently altered in *Adgrb1 *^*exon 2−/−*^ mice that lack FL-BAI1, suggesting a compensatory effect. These isoforms might directly sustain spine development or do so indirectly. For example, if some can bind to the NTF of BAI3, one might hypothesize that they might indirectly rescue spine deficits by enhancing the activation of this closely related receptor that also regulates spinogenesis [5]. It will be of interest to examine whether acute transient knockdown of BAI1 with shRNAs also leads to a similar reprogramming of isoform expression.

G-protein-coupled receptors (GPCRs) are the largest family of membrane-bound receptors and are the targets of ~ 35% of clinically approved drugs [61]. BAI1 is a member of class B GPCRs that are involved in multiple physiological processes including brain development, inflammation, phagocytosis, and diseases such as neurological disorders and tumorigenesis [10, 17, 62]. A full characterization of all isoforms of BAI1 will foster a better understanding of this interesting receptor’s role in health and disease and evaluate its potential as a drug target. Identifying disease-associated isoforms and distinguishing uniqueness in their structures will be critical to target the relevant ones and avoid collateral side effects on others.

## Supplementary Information

Below is the link to the electronic supplementary material.

Supplementary file1 (DOCX 423 KB)

Supplementary file2 (TIF 43079 KB)

Supplementary file3 (TIF 35713 KB)

## Data Availability

Data is available on request.
