# Supplementary material for: Novel Isoforms of Adhesion G Protein-Coupled Receptor B1 (ADGRB1/BAI1) Generated from an Alternative Promoter in Intron 17
Source: Mol Neurobiol. 2024 Jun 28;62(1):900–17. doi: 10.1007/s12035-024-04293-3 (PMC11711277; doi:10.1007/s12035-024-04293-3)
Supplement: Supplementary file 1 — Supplementary file1 (DOCX 423 KB) [file 12035_2024_4293_MOESM1_ESM.docx]

**Supplementary information**

**Supplementary Materials and Methods:**

**Immunoblotting**

Proteins were extracted from cells with Pierce RIPA Buffer (Thermo Fisher Scientific, 89901) or M-PER Mammalian Protein Extraction Reagent (Thermo Scientific, 78501), supplemented with Halt protease and phosphatase inhibitor (Thermo Fisher Scientific, 78442). After extraction, the cell lysates were cleared from debris by centrifugation at 12,000 rpm at 4°C for 15 min. For the conditioned medium, supernatant was collected from culture dishes and filtered with Amicon Ultra-15 centrifugal filter (Merck Millipore, UFC901024). Proteins from the filtered media were then precipitated using acetone and re-dissolved in RIPA buffer. Protein concentrations were determined using a Pierce BCA Protein Assay Kit (Thermo Fisher Scientific, 23225). After that, 1/3 volume of 4x Laemmli Sample Buffer (Bio-Rad, 1610747) supplemented with 10% β-Mercaptoethanol (Sigma, M6250-250), was added to the lysates. Equal amounts of proteins (15-20 ug) were loaded on 10% SDS-PAGE gels and size-separated by electrophoresis with Precision Plus Protein Dual Color Standards (Bio-Rad,161-0394) and transferred to PVDF membranes (Bio-Rad, 1620177) at 300 mAmp for 2 hours. The membranes were blocked with 5% skim milk powder in TBS (Alfa Aesar, J60877.K2) with 0.1% Tween (Fisher, BP337-500) at room temperature for 1 hour, and blotting was performed by incubating the membranes with primary antibodies (1:500- 1:1000 dilutions) at 4°C overnight. Secondary antibodies were then incubated with the membranes at room temperature for 1 hour. Detection was performed with SuperSignal West Pico Plus (Thermo Fisher Scientific, 34580) on the G:Box digital camera system (SYNGENE).

**Gene-specific primers for RT-PCR**

The following primer sets were used to detect exon-specific expression: mouse Adgrb1 (exon 2) F; 5ʹ-tcttcggctacttctcggcggc-3ʹ, R; 5ʹ-cctgtagaaaggcgaggggtgc-3ʹ, (exon 3-6) F; 5ʹ-aggtggctggaaactgtggtccttgtg-3ʹ, R; 5ʹ-tccagggtgaccactcatcccaggc-3ʹ, (exon 7-10) F; 5ʹ-aacgaatggtccagctggag-3ʹ, R; 5ʹ-ggtagtcgatggagacgcag-3ʹ, (exon 12-17) F; 5ʹ-caacctgctggcagaagaga-3ʹ, R; 5ʹ-caggtctggttggtagtgcc-3ʹ, (exon 18-24) F; 5ʹ-cgcgaccatggataaggtga-3ʹ, R; 5ʹ-gttcaccagcacaactgcag-3ʹ, (exon 23-28) F; 5ʹ-tggtgaacatggtgatcggg-3ʹ, R; 5ʹ-tggacacattagctggcagg-3ʹ, (exon 28-31) F; 5ʹ-ctgatgccagcttccctacc-3ʹ, R; 5ʹ-ggtcaatgatgtcctggcca-3ʹ. To detect the alternative first exon, we used transcript 44/2 (new Exon 01) F; 5ʹ-tgacgctgtctcccgtc-3ʹ; transcript 1/81 (exon 18) R; 5ʹ-taagatggcgaaggtggagagc -3ʹ; and (exon 23) R; 5ʹ-gtcccacgaaggcatacagaag -3ʹ.

The genomic DNA sequence located on the 3ʹ end of intron 17 of the human ADGRB1 gene was amplified by PCR with the following primers: XhoI-2210(in17) F; 5ʹ-cgcgtgctagctcgaggtctgcaagggactgtcagaga-3ʹ, XhoI-1190(in17) F; 5ʹ-cgcgtgctagctcgagagtgctgctgctgagggagattga-3ʹ and HindIII-17(in17) R; 5ʹ- ccggaatgccaagctttcatctcctgggacagacacag-3ʹ.

**Graphical representation of BAI1:**

The schematic representation of the domains and motifs of BAI1 was adapted from “GPCR (schematic)”, by BioRender.com (2023). Retrieved from https://app.biorender.com/biorender-templates.

**Supplementary Tables:**

| **Table S1: Information of *Adgrb1* transcripts in the cortex of the mice brain** | | | | | | | |
| --- | --- | --- | --- | --- | --- | --- | --- |
| Transcript ID of *Adgrb1* | Number of independent full length reads supporting this transcript structure | Transcription starts site (TSS)  (in reference to *Adgrb1.1*) | Location of start codon  (in reference to *Adgrb1.1*) | New/short/ extended exons  (in reference to *Adgrb1.1*) | Transcripts length  (in base pairs - bp) | Protein length  (in Amino Acids - aa) | Expected protein size (in kDa) |
| *Adgrb1.1* | 81 | Exon 1 | Exon 2 |  |  | 1582 aa | **173.3** |
| *Adgrb1.3* | 4 | Exon 1 | Exon 2 | Short exon 7.  New exon in intron 24. | 6066 bp | 1612 aa | **176.3** |
| *Adgrb1.4* | 11 | Exon 1 | Exon 2 | Extended exon 27. | 5991 bp | 1587 aa | **173.9** |
| *Adgrb1.5* | 4 | Exon 1 | Exon 2 | Extended exon 27. | 5940 bp | 1602 aa | **175.2** |
| *Adgrb1.9* | 2 | Exon 2 | Exon 2 (different start codon) | Short exon 2.  New exon in intron 24.  Extended exon 27. | 5335 bp | 1537 aa | **168.1** |
| *Adgrb1.12* | 3 | Intron 2 | Alternative first exon in intron 2 | New exon 1 in intron 2. | 4650 bp | 1325 aa | **145.8** |
| *Adgrb1.31* | 9 | Intron 17 | Exon 19 | New exon 1 in intron 17. | 2919 bp | 642 aa | **70.5** |
| *Adgrb1.32* | 2 | Intron 17 | Exon 19 | New exon 1 in intron 17. | 933 bp | 193 aa | **21.3** |
| *Adgrb1.33* | 3 | Intron 17 | Exon 19 | New exon 1 in intron 17.  Extended exon 27. | 2799 bp | 662 aa | **72.3** |
| *Adgrb1.34* | 5 | Intron 17 | Exon 25 | New exon 1 in intron 17.  Short exon 25. | 2639 bp | 401 aa | **43.9** |
|  |  |  | Exon 19 |  |  | 207 aa | **22.8** |
| *Adgrb1.35* | 3 | Intron 17 | Exon 28 | New exon 1 in intron 17.  Exon extension from exon 26 to exon 28 (forms a new single exon). | 6482bp | 319 aa | **34.8** |
|  |  |  | Exon 19 |  |  | 317 aa | **35** |
| *Adgrb1.36* | 49 | Intron 17 | Exon 19 | New exon 1 in intron 17.  Extended exon 27. | 2761 bp | 647 aa | **71** |
| *Adgrb1.37* | 27 | Intron 17 | Exon 28 | New exon 1 in intron 17.  Exon extension from exon 26 to exon 27 (forms a new single exon). | 4107 bp | 319 aa | **34.8** |
|  |  |  | Exon 19 |  |  | 317 aa | **35** |
| *Adgrb1.39* | 6 | Intron 17 | Exon 19 | New exon 1 in intron 17.  New exon in intron 24. | 2816 bp | 675 aa | **73.7** |
| *Adgrb1.40* | 8 | Intron 17 | Exon 19 | New exon 1 in intron 17.  New exon in intron 24.  Extended exon 27. | 2831 bp | 680 aa | **74.3** |
| *Adgrb1.38* | 142 | Intron 17 | Exon 19 | New exon 1 in intron 17. | 2738 bp | 642 aa | **70.5** |
| *Adgrb1.41* | 5 | Intron 17 | Alternative first exon in Intron 17 | New exon 1 in intron 17.  Short exon 18. | 2699 bp | 700 aa | **76.4** |
| *Adgrb1.42* | 19 | Extended Exon 18 | Exon 19 | Extended exon 18. | 2777 bp | 642 aa | **70.5** |
| *Adgrb1.43* | 7 | Extended Exon 18 | Exon 19 | Extended exon 18.  Extended exon 27. | 2733 bp | 647 aa | **71** |
| *Adgrb1.44* | 2 | Extended Exon 18 | Exon 19 | Extended exon 18.  Extended exon 27. | 2778 bp | 662 aa | **72.3** |
| *Adgrb1.54* | 3 | Short Ex24 | Exon 28 | Short exon 24.  Short exon 25.    Exon extension from exon 26 to exon 27 (forms a new single exon). | 4107 bp | 319 aa | **34.8** |
|  |  |  | Exon 25 |  |  | 76 aa | **8.5** |

| **Table S2: Information of *ADGRB1* transcripts in the cortex of the human brain** | | | | | | | |
| --- | --- | --- | --- | --- | --- | --- | --- |
| Transcript Id of *ADGRB1* | Number of independent full length reads supporting this transcript structure | Transcription starts site (TSS)  (in reference to *ADGRB1.1*) | Location of start codon  (in reference to *ADGRB1.1*) | New/short/extended exons  (in reference to *ADGRB1.1*) | Transcripts length  (in base pairs - bp) | Protein length  (in Amino Acids - aa) | Expected protein size  (in kDa) |
| *ADGRB1.1* | 2 | Exon 1 | Exon 2 |  | 5893 bp | 1584 aa | **173.5** |
| *ADGRB1.4* | 2 | Extended exon 9 | Exon 10 | Extended exon 9 | 3608 bp | 941 aa | **103.8** |
| *ADGRB1.7* | 3 | Intron 17 | Alternative first exon in Intron 17 | New exon 1 in intron 17 | 2839 bp | 699 aa | **76.9** |
| *ADGRB1.8* | 22 | Intron 17 | Exon 19 |  | 2727 bp | 644 aa | **70.8** |
| *ADGRB1.15* | 2 | Exon 27 | Exon 28 | Short exon 27  New exon in intron 27 | 1801 bp | 213 aa | **23.4** |
| *ADGRB1.18* | 14 | Extended exon 28 | Exon 28 | Extended exon 28 | 1802 bp | 213 aa | **23.4** |
| *ADGRB1.14* | 2 | Short exon 25 | Exon 25 | Short exon 25  New exon in intron 27  Extended exon 31 | 1939 bp | 449 aa | **49** |
| *ADGRB1.16* | 2 | Short Exon 27 | Exon 28 | Short exon 27  New exon in intron 27  Extended exon 31 | 1804 bp | 214 aa | **23.5** |
| *ADGRB1.19* | 17 | Extended exon 28 | Exon 28 | Extended exon 28 | 1802 bp | 213 aa | **23.4** |
| *ADGRB1.20* | 2 | Short exon 28 | Exon 28 | Short exon 28  Exon extension from exon 30 to exon 31 (forms a new single exon). | 2000 bp | 223 aa | **23.9** |

**Supplementary Figures:**

**FIGURE S1**

**A)** Impact of boiling on BAI1 detection by western blot. Protein was extracted by using RIPA buffer and M-PER. Boiling of protein at 95 °C for 1 min or 5 min decrease the amount of detectable BAI1 when probed with C-BAI1 [b] antibody (epitope 1537-1567aa). In contrast, to detect pERK1/2 (T202/Y204) boiling is required for 1-5 mins. There is no impact of boiling on GAPDH detection.

**B)** Expression of BAI1 isoforms in cerebrum and pituitary of WT and exon 2 deleted (KO) C57BL/6J mice by using C-BAI1 [b] antibody (epitope 1537-1567aa).

**C)** Transient transfection of *ADGRB1* cDNA in medulloblastoma cell lines reveals the major FL-BAI1 ~200 kDa isoform and its cleavage products of ~70 kDa and ~75 kDa. C-BAI1[b] antibody was used.

**FIGURE S2:** Structure of *ADGRB1* mRNA variants detected in human breast tumors by analyzing long-read RNA-seq data.

**A)** Full length (FL)- *ADGRB1* transcript is shown on the top and IDs of individual transcripts (total eight) is indicated on the right. The left side box shows an enlarged view of the new exons used by transcripts starting within a ~1000 bp region encompassing exon 18 (in ~1000bp range). Exons are represented by solid boxes, introns by a line.

**B**) Top: New exons 1 (pink rectangles) of transcript variants of *ADGRB1* starting in 3ʹ end of intron 17 (5 out of 8) identified in human breast tumor. One transcript starts within exon 18 and another from a new exon in intron 18. Length of the new exon 1, splicing to exon 18 and start codons (ATG) for same open reading frame (ORF) as the full length *ADGRB1* are indicated.

Bottom: Structure of hBAI1 isoforms generated from variant mRNAs containing a translation start site in new exons 1 and exon 19. Two transcripts (PB.34262.1 and PB.3426.3) are transcribed from new exons 1 located at the 3′end of intron 17. They have overlapping 5ʹ UTR but the same coding sequence with an ATG in their new exon 1. Splicing in between new exon 1 and exon 18 generates proteins that are in frame with the main reading frame of FL-hBAI1.These transcripts encode a hBAI1 isoform with a 60aa N-terminus that has a GPS site but no GAIN domain and are thus not expected to be cleaved by autoproteolysis. Of note, the amino acid sequence of the N-terminus only partially overlaps with the FL-hBAI1 region. The 7 N-terminal amino acids in white circles derive from translation of new codons in new exon 1 that do not exist in FL-BAI1 in human. The other 5 transcripts are being translated from exon 19 and generate identical BAI1 isoforms with 5aa long N-termini.

**WT**

**KO**

**Cerebrum**

**Pituitary**

**Mouse**

**WT**

**KO**


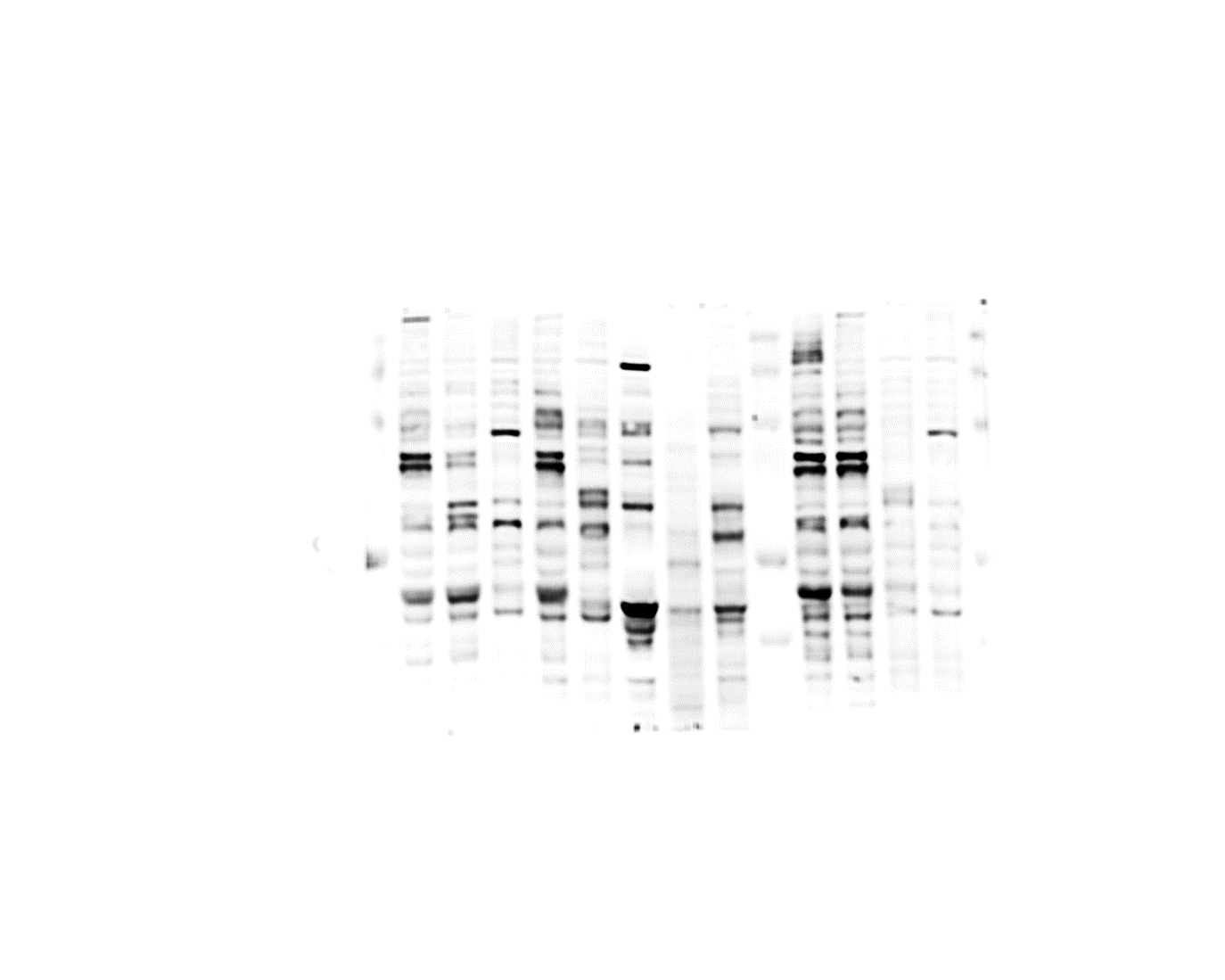


**BAI1**(C-term)

**- 50**

**- 75**

**- 150**

**- 100**

**- 250**

**- 37**


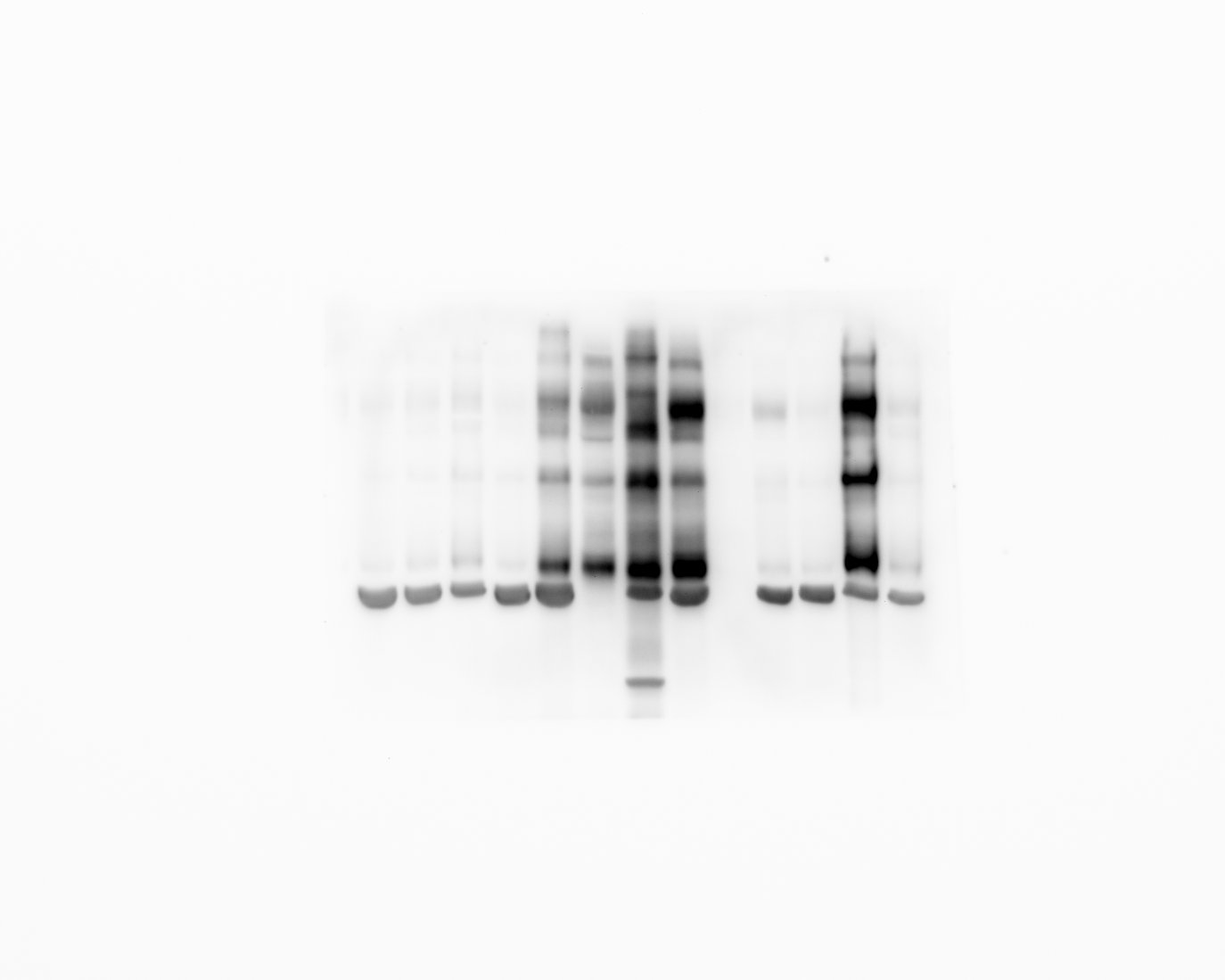


**β-Actin**

**- 50**
